# Using quantitative features extracted from T2-weighted MRI to improve breast MRI computer-aided diagnosis (CAD)

**DOI:** 10.1371/journal.pone.0187501

**Published:** 2017-11-07

**Authors:** Cristina Gallego-Ortiz, Anne L. Martel

**Affiliations:** 1 Department of Medical Biophysics, University of Toronto, Ontario, Canada; 2 Physical Sciences, Sunnybrook Research Institute, Toronto, Ontario, Canada; University of Chicago, UNITED STATES

## Abstract

Computer-aided diagnosis (CAD) has been proposed for breast MRI as a tool to standardize evaluation, to automate time-consuming analysis, and to aid the diagnostic decision process by radiologists. T2w MRI findings are diagnostically complementary to T1w DCE-MRI findings in the breast and prior research showed that measuring the T2w intensity of a lesion relative to a tissue of reference improves diagnostic accuracy. The diagnostic value of this information in CAD has not been yet quantified. This study proposes an automatic method of assessing relative T2w lesion intensity without the need to select a reference region. We also evaluate the effect of adding this feature to other T2w and T1w image features in the predictive performance of a breast lesion classifier for differential diagnosis of benign and malignant lesions. An automated feature of relative T2w lesion intensity was developed using a quantitative regression model. The diagnostic performance of the proposed feature in addition to T2w texture was compared to the performance of a conventional breast MRI CAD system based on T1w DCE-MRI features alone. The added contribution of T2w features to more conventional T1w-based features was investigated using classification rules extracted from the lesion classifier. After institutional review board approval that waived informed consent, we identified 627 breast lesions (245 malignant, 382 benign) diagnosed after undergoing breast MRI at our institution between 2007 and 2014. An increase in diagnostic performance in terms of area under the curve (AUC) from the receiver operating characteristic (ROC) analysis was observed with the additional T2w features and the proposed quantitative feature of relative T2w lesion intensity. AUC increased from 0.80 to 0.83 and this difference was statistically significant (adjusted p-value = 0.020).

## Introduction

Breast magnetic resonance imaging (MRI) is currently the imaging modality with the highest sensitivity for detecting breast cancer in high-risk women and plays a significant role in evaluating the extent of disease in newly diagnosed breast cancer [1]. In 2011 the Ontario Breast Screening Program (OBSP) launched the high-risk screening program and started offering annual breast MRI in addition to mammography to women at high risk of developing breast cancer. Women are eligible if they have no acute breast symptoms, are 30 to 69 years of age and meet at least one of the personal and family history risk criteria [2].

With breast MRI, breast lesions are characterized based on the uptake of gadolinium contrast agent during a T1-weighted (T1w) dynamic contrast-enhanced (DCE-MRI) sequence, while T2-weighted (T2w) MRI is routinely used to rule out the presence of cysts, intra-mammary lymph nodes and other benign breast findings [3]. Evaluation of breast MRI findings is typically performed by visual assessment of contrast enhancement kinetics and morphology using diagnostic descriptors established by the American College of Radiology (ACR) Breast Imaging Reporting and Data System (BI-RADS) lexicon [4]. The key challenge in breast MRI is that despite standardization guidelines, evaluation suffers from high inter-observer variability [5]. In addition, breast MRI requires considerable time for image processing and interpretation. Computer-aided diagnosis (CAD) has been proposed as a tool to standardize evaluation, to automate time-consuming analysis, and to aid the diagnostic decision process by radiologists [6].

In clinical practice it is widely recognized that T2w MRI findings are diagnostically complementary to T1w DCE-MRI findings [3, 7, 8] and while CAD has been extensively studied for DCE-MRI aided interpretation [9], few studies have examined the complementary role of T2w MRI lesion characterization in CAD. Bhooshan et. al. [10] found that T2w texture offered additional discrimination between malignant and benign lesions, but T2w signal intensity (i.e absolute intensity) had poor discrimination. Visual assessment of T2w MRI findings, however, resembles more closely the analysis of lesion intensity with respect to an adjacent tissue intensity (i.e relative intensity). In fact, the BI-RADS lexicon defines T2w lesion appearance using three categories: hypointense or not seen, slightly hyperintense, and hyperintense. Ballesio et. al. [11] investigated the ratio of the lesion to the muscle intensity, which reflects more faithfully hyperintensity or hypointensity of a lesion, called the lesion-to-muscle signal intensity ratio (LMSIR), as an adjunct feature to other BI-RADS diagnostic descriptors. The study found that LMSIR improved the differential diagnosis of benign and malignant lesions in borderline BI-RADS categories 3 and 4. Consequently, a T2w CAD feature that reflects hyperintensity or hypointensity with respect to adjacent or reference tissue can offer additional discrimination between malignant and benign lesions and improve CAD performance. The method described in [11] to measure LMSIR requires manual selection of a region over the pectoralis muscle to measure a reference intensity, making relative T2w lesion intensity difficult to include into an automated CAD system. In this work, we propose an automated feature of relative T2w lesion intensity using a predictive regression model. The primary aim of this study was to incorporate relative T2w lesion intensity and texture to more standard DCE-MRI features and to investigate the added value of T2w-based features in the task of distinguishing breast malignant from benign lesions.

The assistance of CAD in screening breast MRI as part of clinical routine is still an area of ongoing research, but some pilot studies have shown benefits. Meinel et. al. [12] found that performance of human readers (regardless of experience) improved significantly when aided by a CAD system. Improving the predictive performance of CAD for breast MRI as we show in this work is, therefore, an important prerequisite for the applicability of a CAD system to human reading studies.

## Materials

After our institution Sunnybrook Health Sciences Centre research ethics review board (REB) approved and waived informed consent, we retrospectively reviewed breast MRI studies conducted at our institution between 2007 and 2014. We identified histopathology proven breast lesions reported in breast MRI imaging studies performed prior to biopsy or surgery in which ground truth histopathology was obtained (see Table 1). Only women without prior breast surgery or cancer radiotherapy or chemotherapy treatment were considered in this study. We identified 435 women aged 22 (min) to 85 (max), or average age at imaging of 49 ± 10.6 years (mean ± std) with a total of 627 histopathology proven breast lesions (245 malignant, 382 benign). 322 lesions were found in high-risk patients undergoing breast MRI screening. For all lesions, ground truth histopathology diagnosis was classified as benign or malignant based on the diagnostic procedure results: biopsies (n = 568: Core Needle n = 313, Fine Needle Aspiration n = 14, or Vacuum Assisted n = 235, not specified n = 6) or surgery (n = 59) performed at our institution. The median time between imaging and diagnostic procedure was 20 days, interquartile range (IQR) was 11 − 32 days. BI-RADS assessment categories from breast MRI ranged from 2 to 6. Histopathology of lesion BI-RADS 2 and 3 was obtained due to additional imaging such as ultrasound examination or subsequent clinical decision management.

**Table 1:** Patient and lesion histopathology characteristics.

Characteristic	N	(%)
**Patient age**		
<40 years	149	23.8
40-50 years	230	36.7
50-74 years	238	37.9
>74 years	10	1.6
**BI-RADS assessment score**		
BI-RADS 2	21	3.3
BI-RADS 3	57	9.1
BI-RADS 4	343	54.7
BI-RADS 5	116	18.5
BI-RADS 6	90	14.4
**Lesion enhancement type**		
Mass	407	64.9
Non–mass	220	35.1
**Benign lesions**	**382**	**61**
Benign breast parenchyma	72	18.8
Fibrocystic	70	18.3
Fibroadenoma	70	18.3
Atypical Ductal Hyperplasia	34	8.9
Atypical Lobular Hyperplasia	16	4.2
Fibrosis	20	5.2
Duct Papilloma	19	5.0
Sclerosing Adenosis	19	5.0
Columnar cell changes	18	4.7
In-situ Lobular Carcinoma	7	1.8
others	37	9.8
**Malignant lesions**	**245**	**39**
Invasive Ductal Carcinoma	136	55.5
In-situ Ductal Carcinoma	80	32.7
In-situ papillary Carcinoma	3	1.2
Invasive Lobular Carcinoma	21	8.6
others	5	2.0

All women underwent MRI on a 1.5-T magnet (Signa, General Electric Medical Systems, Milwaukee, Wis) using a dedicated breast coil. The imaging protocol included sagittal T2-weighted FSE fat saturated images and simultaneous bilateral sagittal DCE-MRI T1-weighted fat saturated 3D FSPGR images. The dynamic protocol comprised one pre-contrast and four post-contrast acquisitions using a bolus injection of 0.1 mmol/kg of Gadolinium contrast agent Gadovist^®^(gadobutrol) injection at 2 cc/sec, 20-second delay. All DCE-MRI volumes had less than 1 mm in-plane resolution, 2 to 3 mm slice thickness, and under 120 seconds per dynamic acquisition.

## Methods

**Fig 1:**
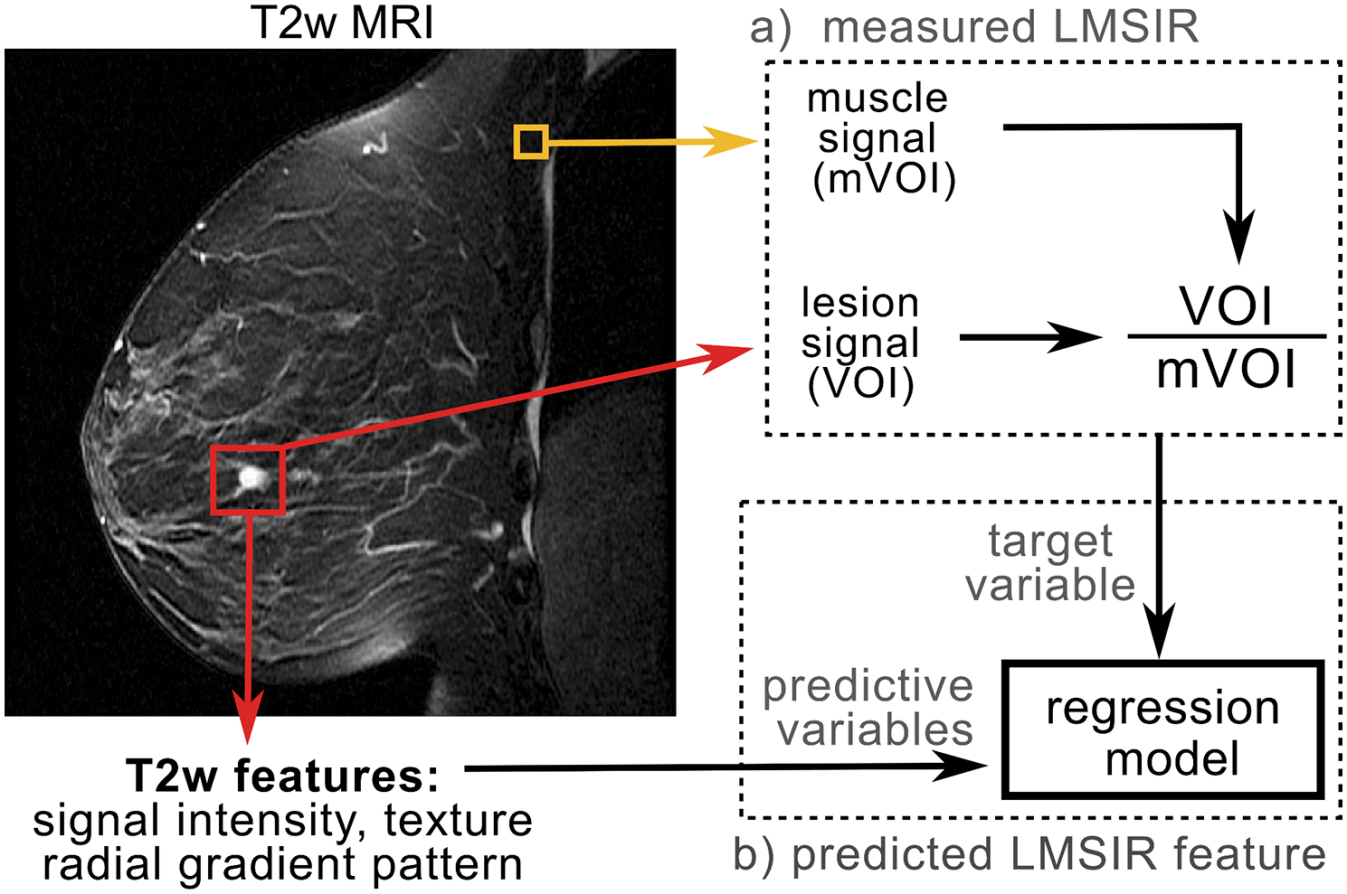
Pipeline for extracting the proposed T2w CAD features. a) Steps involved in measuring Lesion-to-muscle signal intensity ratio (LMSIR). b) steps involved to predict LMSIR from T2w features.

Since all T1w and T2w scans are acquired using the same DICOM reference system, volumes were aligned using patient position and orientation information. None of the studies were affected by significant patient motion determined at the time of the review by the radiologist, so alignment to the same reference system was sufficient. The main steps involved in generating T1w-, T2w-CAD features, and relative T2w lesion intensity or predicted LMSIR are illustrated in Fig 1. Next, we describe each step in more detail.

### Lesion segmentation and generation of image-based features

Lesions were segmented as based on the area of enhancement on T1w DCE-MRI using the location quadrant and clock position from the radiologist report. An interactive seeded 3D region growing algorithm [13] was employed to segment each lesion volume-of-interest (VOI). A total of 197 T1w DCE-MRI derived features comprising 34 kinetic, 19 morphological and 44 texture features were used, with additional 80 relative post-contrast enhancement uptake features (20 measurements per post-contrast volume) and 20 spatial dispersion of signal enhancement features. By overlaying the segmented lesion on the T2w MRI volume, we extracted T2w measurements from the corresponding area of T1w enhancement. T2w MRI derived features consisted of signal intensity, texture and margin morphology for a total of 43 features. Feature categories are briefly described as follows and a more detailed description is given in the supporting information S1 Table:

#### T1w DCE kinetic

Kinetic features capture the dynamic characteristics of enhancement (e.g rates of initial uptake and subsequent washout of contrast agent, time to peak, and curvature at peak enhancement). Other features include the maximum variation of enhancement, the post-contrast time at which the maximum variance occurs, and variance rates, as described in the literature [14, 15]. Kinetic features were obtained from voxels within the lesion and from boundary voxels, by fitting a logarithmic model to the temporal enhancement curve [16, 17]. Analysis was performed for both mass and non-mass lesions since prior work [13] revealed that some kinetic features are discriminative for both groups, with additional kinetic features being selectively relevant for either group but not the other.

#### Dispersion of DCE-MRI

Enhancing voxels within the lesion are clustered according to their spatial proximity. Then, the distance of each cluster to the centroid of the lesion is calculated and averaged over all clusters to derive a dispersion feature. Low dispersion is observed in lesions with more proximal clusters since enhancing voxels occur more closely together. Less proximal clusters indicate higher dispersion since enhancing voxels occur farther apart from one another, as proposed in [13].

#### Lesion morphology

Analysis of 3D lesion morphological characteristics. Radial gradient pattern features quantify whether or not lesion uptake (in T1w) or internal signal-intensity (in T2w) extends in a radial pattern [18]. Margin gradient quantifies changes at the margin of the lesion. Additional morphology features describing the circularity, irregularity, and margin sharpness [19] and the spiculation of lesion borders [20] were extracted from T1w-DCE imaging only.

#### T1w DCE and T2w texture

We implemented 3D texture features based on the nondirectional grey-level co-occurrence matrix (GLCM) of voxel-pair statistics as proposed by [21]. For T1w texture, nondirectional GLCMs were generated from each of the four post-contrast volumes by summing 13 directional GLCMs. Then, from the resulting nondirectional GLCM matrix, different measures (e.g, energy, contrast enhancement, similarity, and correlation) can be derived. Texture features were also derived from the T2w volume.

### Estimating lesion-to-muscle signal intensity ratio (LMSIR)

We proposed a model of relative T2w lesion intensity based on the Lesion-to-muscle signal intensity ratio (LMSIR). Using a rectangular 5 *mm*^3^ box, placed manually in the pectoralis muscle area, we extracted the T2w intensity of the muscle, as proposed in [11]. The manual LMSIR was measured by dividing the intensity average in the T2w lesion VOI by the intensity average in the muscle. Then, a regression model was derived based on ensembles of boosting regression trees using the manual LMSIR as the target variable and T2w-derived features as predictive variables (see Fig 1). To validate the proposed automated feature of relative T2w lesion intensity in an unbiased setting, 10-fold cross-validation was used: At each fold, 9/10 of the patients are split into 80% training and 20% validation sets. The training set is used for finding all relevant variables via Z-scores [22], and the validation set for evaluating the quality of predictions and the optimal number of regression trees. Root mean square error (RMSE), $RMSE=\sqrt{{\left(measured-predicted\right)}^{2}}$ was chosen as a metric for quality of predictions. The number of regression trees with the lowest RMSE in the validation set was used to estimate the relative T2w lesion intensity feature in the independently held-out fold of the remaining one-tenth of the patients.

### Added diagnostic value of T2w features in CAD

CAD lesion classification for differentiating benign and malignant lesions was achieved using ensembles of boosting classification trees. The goal of ensemble learning is simple: to improve the accuracy of a classifier by combining weak classifiers into a committee. We used AdaBoost [23], where decision trees are built sequentially and misclassified cases are emphasized to improve their predictions. As training progresses datasets are weighted accordingly to their misclassification probability. Misclassified instances (difficult classifications) receive higher weights, so that in subsequent trees the emphasis on producing correct classifications is increased with respect to correctly classified instances.

In order to assess the added diagnostic value of T2w features, in particular the discrimination added by the relative T2w signal intensity as opposed to the absolute T2w signal; CAD classification performance was compared between the following distinctive feature spaces:

- *group-1*) only T1w DCE-MRI features,
- *group-2*) combined T1w DCE-MRI, T2w texture, margin, and T2w signal intensity (SI) features,
- *group-3*) combined T1w DCE-MRI, T2w texture, margin, and T2w signal intensity (SI) and measured LMSIR features,
- *group-4*) combined T1w DCE-MRI, T2w texture, margin, and predicted LMSIR features.

### Statistical validation of CAD classification performance

To validate CAD lesion classifiers built with each of the four distinctive feature spaces, we used 10-fold cross-validation: At each fold, 9/10 of the patients are used as 80% training and 20% validation sets and the remaining one-tenth of the patients as an independently held-out test set. The training set was used for AdaBoost model training and the validation set for tuning the optimal number of trees in the final ensemble and the optimal maximum depth of decision trees. Parameters that led to the lowest classification error in the validation set were chosen for the final CAD classifier trained at each fold. Then, to ensure an unbiased assessment of CAD classification performance, the final CAD classifier is used to predict benign or malignant class in the held-out test set. In addition, to avoid the bias of multiple lesion effects, lesions were stratified at the patient level, so that multiple lesions from the same patient were always in separate folds.

The area under the curve (AUC) from the receiver operating characteristic (ROC) analysis was calculated to compare the diagnostic performance of CAD lesion classification built with the different feature spaces. By comparing the AUC of each feature space, the added diagnostic value of T2w features when combined with T1w features and with a feature of relative T2w lesion intensity can be evaluated. To calculate AUCs the pROC package in R [24] was used. The package computes AUCs based on the trapezoid method. Comparison between two AUCs is implemented via the bootstrap percentile method. Given two AUCs *θ*_1_, *θ*_2_, $Z=\frac{\theta_{1}-\theta_{2}}{sd(\theta_{1}-\theta_{2})}$ measures the difference between AUCs, where *sd* is the standard deviation of the difference between AUCs. If *Z* is calculated multiple times, each time with a replicate sample with replacement from the original, the resulting distribution approximately follows a normal distribution, and a two-tailed p-value of the difference in AUC can be calculated from the resampled Z distribution. For AUC difference hypothesis testing a p-value of ≤0.05 was considered significant and a Bonferroni correction was added, in which the p-values are multiplied by the number of comparisons, to adjust for multiple comparisons across the four feature groups. The bootstrap resamples were stratified so that the same number of observations per class in the original sample is kept in each resample.

### Assessing complementary role of T2w features to T1w features

To assess the complementary role of T2w features to T1w based characterization of lesions, it is important to quantify the contribution of each individual feature. A surrogate of feature contribution to prediction output in ensembles of tree classifiers is the number of times a feature is selected within the trees. In this sense, the most contributing features can be identified as those which are consistently selected across multiple trees built with different samples of the data. This is possible using a re-sampling technique such as cross-validation. Following this rationale, “very contributing” features can be defined as features selected in more than 75% of the sampling folds, “moderately contributing” features as those selected in 50-to-75% of the folds, while “low contributing” features as those selected in less than 50% of the folds.

### Explaining CAD classification via feature rules

Intuitively, our CAD classifier uses a set of decision rules to classify lesions into benign or malignant classes according to the characteristics of each class. Next we explain how decision rules can be extracted from the final ensemble of decision trees to explain CAD classification results.

The key idea behind decision rule extraction from trees is recursion. A tree is built by recursively splitting a set of observations at the root node **S** into subsets **S**_**j**_ using feature-value splits. If every observation in **S**_**j**_ is of the same class or if **S**_**j**_ is very small, recursive splitting stops and the node becomes a leaf node. Leaf nodes predict a lesion class according to the most frequent class of observations at the node. Rules can then be extracted by traversing the resulting tree. Binary splits from the root to a leaf node grouped together define the condition of the rule **C**, and the predicted class assigned at the leaf node the outcome of the rule **T**. Rule statements {**C** ⇒ **T**} can be formed to explain CAD classification results. These rules elucidate the most discriminative features used by the classifier to assign individual observations to classes. These most discriminative features are not BI-RADS classifications, instead they are automated features used by the CAD classifier that reflect kinetic, morphology, dispersion or texture properties of lesions. A perfect classification rule makes no mistakes in mapping observations to classes, but since observations at the leaf nodes may not entirely be of one class, classification rules are imperfect and produce some classification errors.

Top scoring rules are those with low error rate and high popularity or high frequency of occurrence. Frequency measures the fraction of observations that satisfy a given rule condition, and error rate the fraction of observations that are misclassified by the given rule condition. Consequently, classification rules can be ranked accordingly to error rate and frequency in order to determine the top scoring rules that explain a given CAD classification. In this work, we used the simplified tree ensemble learner (STEL) framework [25] to extract rules and measure rule quality based on frequency and error rate. Top scoring rules were defined as those with less than 10% error rate and a minimum frequency of occurrence in ten observations. We employed the top five scoring to explain CAD lesion classification results.

To increase the interpretability of extracted rules, we categorized features splits with thresholds. For a numerical feature *x* ∈ *X*, a node split *x* ≤ *θ*_*j*_ can be mapped into the corresponding quantile level of the feature, according to its univariate probability density function *Pr*[*x* ≤ *θ*_*j*_]. We transformed feature quantiles: 0%(min), 25%(Q1), 50%(Median or Q2), 75%(Q3) and 100%(max), to ordinal categories: very low, low, medium, high and very high. As a result, feature values associated with node splits can be transformed to ordinal categories for human-readable categorization of feature split conditions. Furthermore, rule extraction and ranking can help elucidate the additional contribution of T2w features when combined with T1w features. For example, CAD lesion characterization with additional T2w features can enhance differences between certain benign and malignant lesions and improve the overall performance of a CAD system. We used extracted rules to explain some CAD lesion classification results and illustrate combinations of T1w and T2w features that enhance lesion classification.

## Results

### Predictive model of T2w relative signal intensity

Muscle intensity from manually placed regions (mVOI) had a median value of 45, inter-quartile range (IQR) [32–61] absolute intensity values. The variability of the pectoralis muscle intensity was compared between benign and malignant cases and an independent t-test confirmed that the muscle is a reliable tissue of reference as it does not vary significantly between these groups (t = 0.002, p-value = 1).

**Fig 2:**
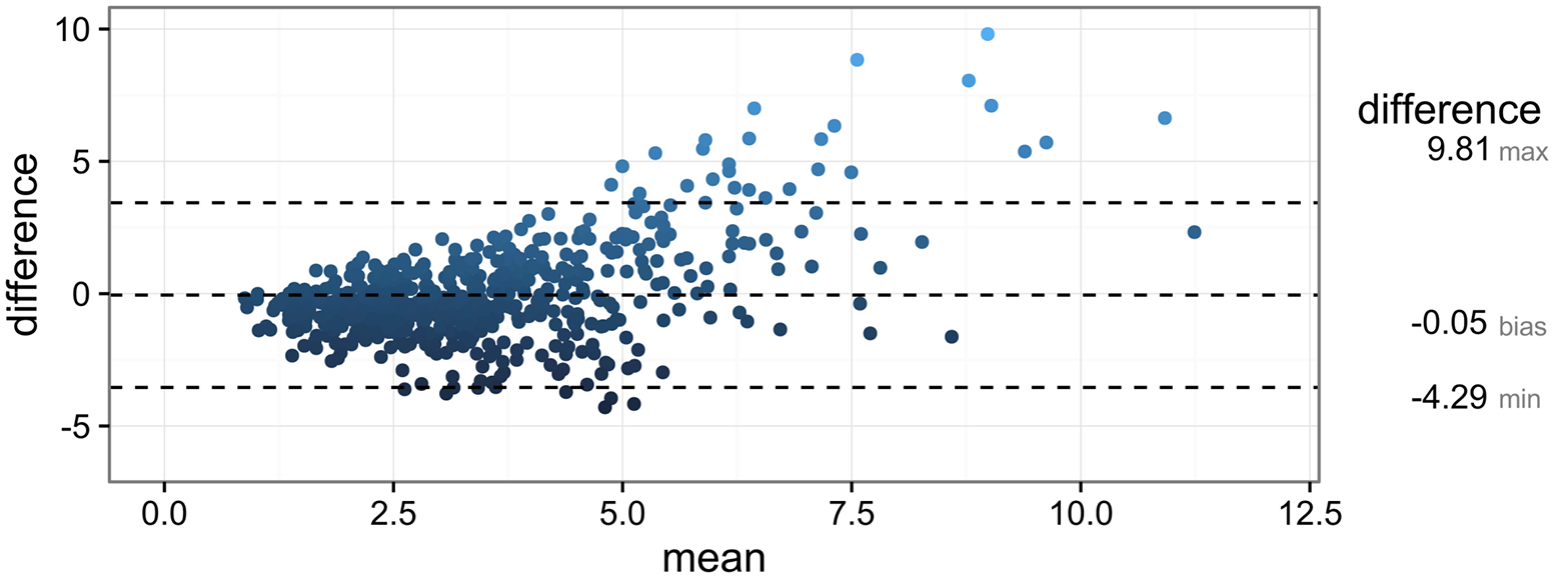
Bland-Altman plot of the agreement between measured and predicted LMSIR as a CAD feature of relative T2w lesion intensity.

Measured LMSIR had a median value of 2.84, IQR [1.92 − 4.17]. Predicted LMSIR from the regression model had a median value of 3.24, IQR [2.53 − 4.10]. The median RMSE of the predicted LMSIR across all cross-validation folds was 0.89 IQR: [0.47-1.58]. A Bland-Altman plot of the agreement is shown in Fig 2. Overall, there was not a significant systematic difference, bias = -0.053, limits of agreement (average difference ±1.96 standard deviation of the difference) = [-3.54, 3.44]) but differences were higher at high values of measured LMSIR (mean LMSIR over 7.5). Only one outlier had (measured-predicted LMSIR > 10) or difference LMSIR = 20.6 (point not shown). The lesion was a benign lesion presenting as nonmass segmental enhancement interposed between several clustered cysts. However, this discrepancy in relative T2w lesion intensity (measured LMSIR = 26.8, predicted LMSIR = 6.6), did not affect the correct prediction of low probability of malignancy by the CAD lesion classifier.

### CAD performance according to feature groups

Performance was highest when combining T1w DCE-MRI with T2w texture and margin features and predicted LMSIR (*group-4*), diagnostic accuracy was AUC = 0.83, 95% CI [0.80-0.87]. Performance was similar for classifiers in *group-3*, AUC = 0.82 95% CI [0.78-0.85], and slightly lower when combining T1w DCE-MR with T2w texture, margin, and absolute signal intensity (SI) (*group-2*), AUC was 0.81, 95% CI [0.77-0.84]. Diagnostic accuracy of only conventional DCE-T1w features (*group-1*) was the lowest, AUC = 0.80 95% CI [0.76-0.84]).

**Fig 3:**
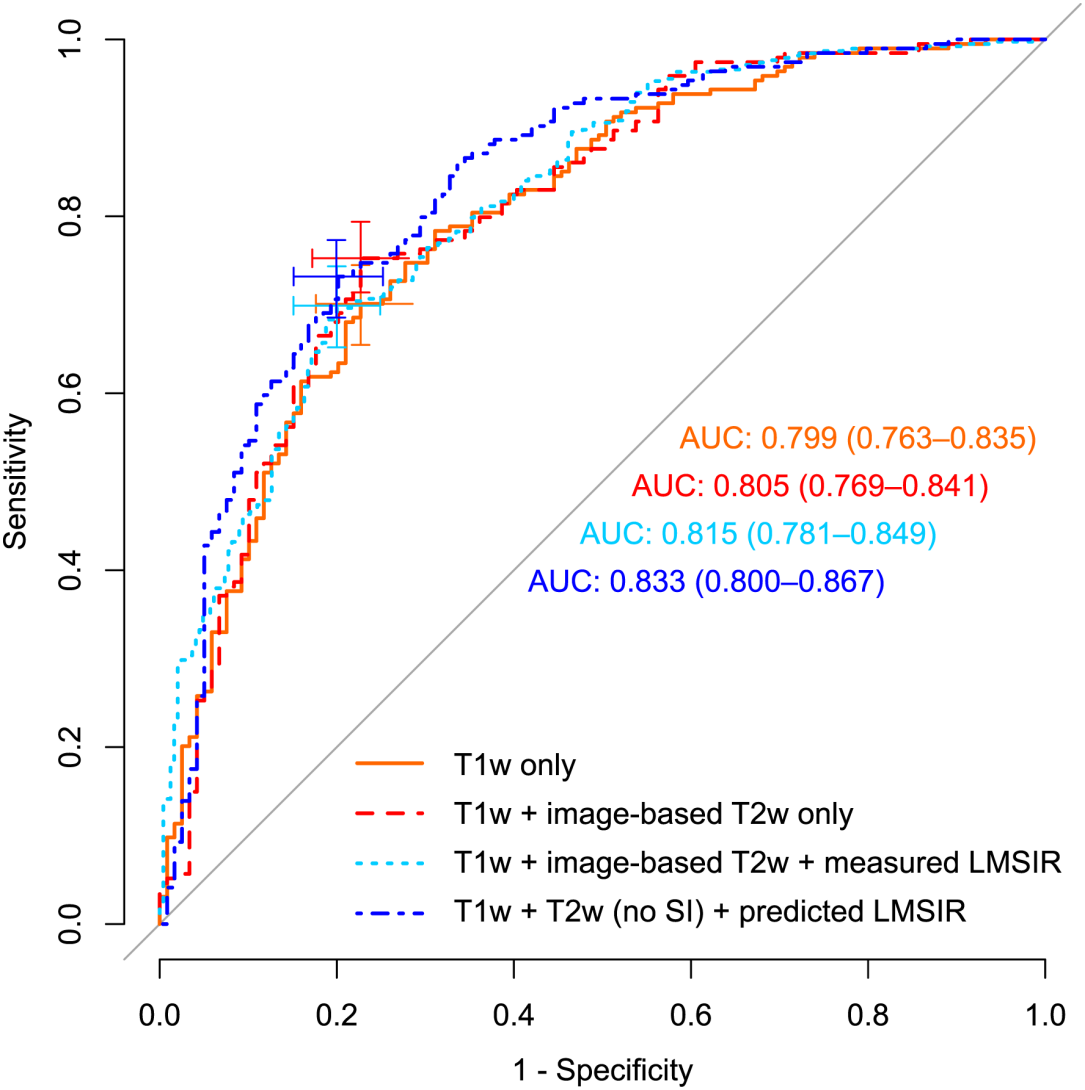
Receiver operating characteristic (ROC) and Area Under the Curves (AUC)—95% confidence-interval (CI) for each feature space. Cross marks indicate optimal operating point (OP) and 95% CI of Sensitivity and Specificity.

An maximum increase of 3.4% in AUC with additional T2w texture, margin and predicted LMSIR features was observed, compared to ensembles with only DCE-T1w features. Fig 3 and Table 2 show diagnostic performance results of the four feature space classifiers based on held-out datasets pooled from all cross-validation folds. Table 3 reports the adjusted p-values of AUC differences across groups. AUC differences between *group-1* and *group-4* were the only statistically significant (adjusted p-value = 0.020). Differences in performance between *group-1* and feature groups with only image-based T2w features (i.e *group-2*), and with measured LMSIR (i.e *group-3*), were not statistically significant (see Table 3).

**Table 2:** CAD performance according to feature groups. Sensitivity and Specificity are reported at the optimal operating point of group 1 in ROC space.

Feature group	AUC	Sensitivity	Specificity
1. only T1w DCE-MRI	0.80	0.70	0.75
2. T1w, T2w-(texture, margin, SI)	0.81	0.70	0.76
3. T1w, T2w-(texture, margin, SI), measured LMSIR	0.82	0.70	0.76
4. T1w, T2w-(no SI) with predicted LMSIR	0.83	0.70	0.80

**Table 3:** Bonferroni corrected p-values of AUC performance differences across groups.

adj. p-value	*group-1*	*group-2*	*group-3*	*group-4*
*group-1*	—-	1.00	1.00	0.020 *
*group-2*	—-	—-	1.00	0.079
*group-3*	—-	—-	—-	1.00
*group-4*	—-	—-	—-	—-

Using the sensitivity achieved by DCE-T1w only classifier at the optimal operating point of 0.70 as a reference sensitivity, the achieved specificities by each of the groups were: 0.75 for (*group-1*), 0.76 for (*group-2*), 0.76 for (*group-3*), and 0.80 for (*group-4*). Therefore, the highest predictive performance achieved for CAD classification of benign and malignant lesions was achieved by the combination of T1w and T2w features (without absolute SI) but with predicted LMSIR and had a sensitivity of 0.70, and specificity of 0.80.

### Assessing contribution of T2w features to classification output

Tree-based feature relevance analysis confirmed that T2w features contributed to the predictive outcome of classifiers that combined DCE-T1w with additional T2w features. Group-4 classifier used frequently T2w texture, margin and predicted LMSIR features available, 21 features were selected in 75% or more of the sampling folds, and only one texture feature in 50-to-75% of the folds. This indicates a consistent contribution of T2w features. T2w features selected with high frequency included: 10 texture features and 10 T2w radial margin gradient pattern features. Predicted LMSIR was also selected with high frequency (in all 10 folds).

### Explaning CAD classification via feature rules

Rules scoring according to our top scoring threshold were extracted for each lesion in the left-out fold during cross-validation. CAD classification outputs were explained by top five scoring rules. 72% of lesions (450/627: 292 benign and 158 malignant) were explained by at least one T2w feature among the top five rule conditions, whereas 28% of lesions (177/627: 90 benign and 87 malignant) were explained by only T1w features among the top five rule conditions.

Features offered selective explanations according to pathology. For example, relative T2w lesion intensity predicted with our regression model was present in the top five scoring rules that explained 0.357 (25/70) of all Fibrocystic cases but only explained 0.0662 (9/136) of invasive ductal carcinoma cases.

**Fig 4:**
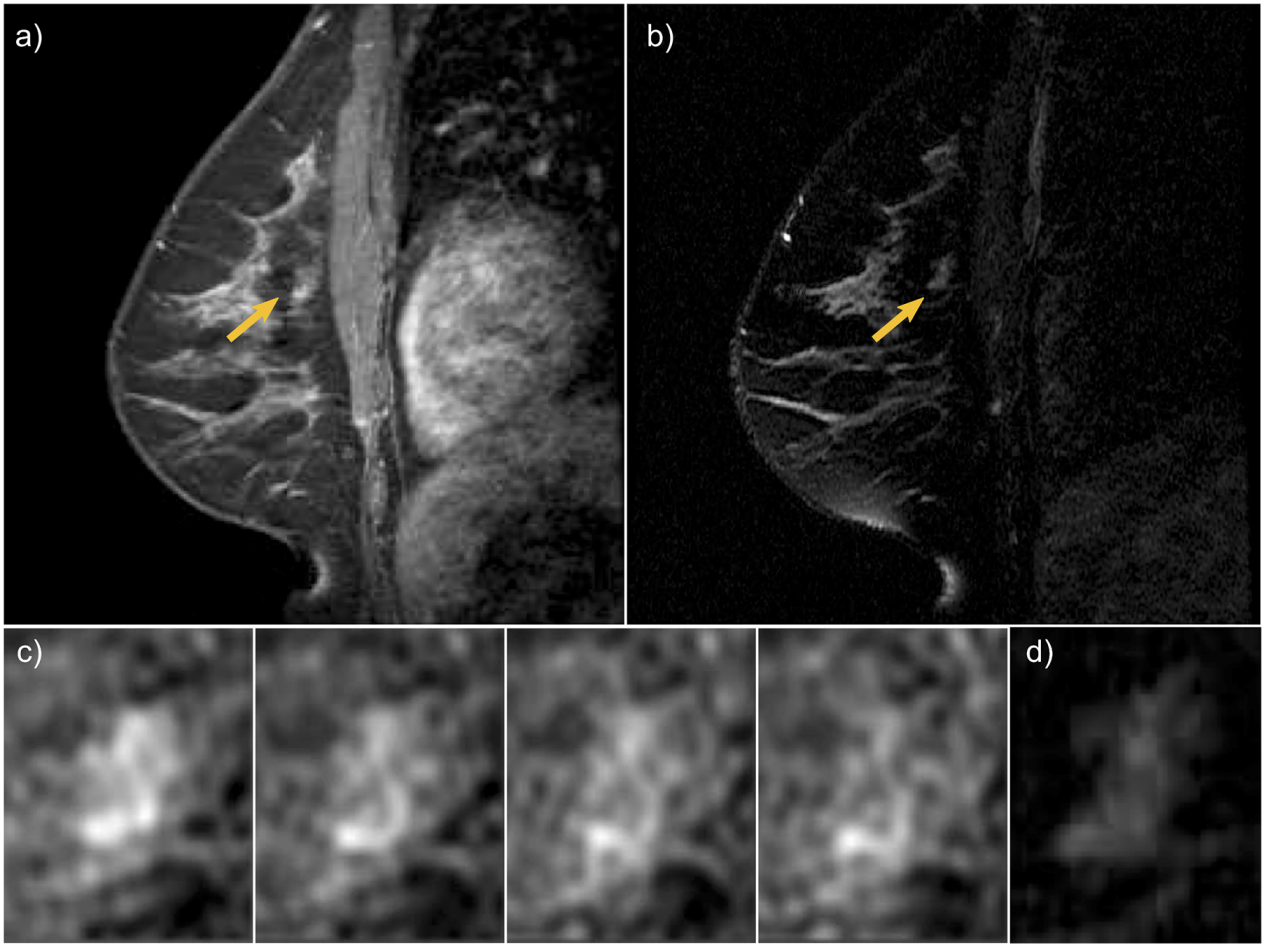
Invasive ductal carcinoma in a 36-year old high risk woman appeared as a focal non-mass, with persistent enhancement (BI-RADS score 4). a) T1w 4th post-contrast DCE-MRI. b) T2w MIR. c) zoomed progressive enhancement at each of the four post-contrast scans. d) zoomed lesion T2w appearance.

Fig 4 shows an invasive ductal carcinoma, a malignant lesion in a 36-year old high-risk woman BRCA2 mutation carrier. The lesion appeared as a focal non-mass, with persistent enhancement (BI-RADS score 4). In Table 4 we report the top two scoring rules that explained CAD lesion classification as a malignant lesion. Rule metrics in the first two columns indicate the frequency and error rate observed when the rule was extracted from the original ensemble. Frequency is the fraction of lesions in our database that satisfied the rule condition, and error rate the fraction of lesions misclassified by the given rule condition. The condition of the rule and type, the probability of malignancy and predictive outcome associated with the rule are provided in the other columns respectively.

**Table 4:** Explanation of CAD classification output for lesion in Fig 4.

Freq	Err	condition	Pred	Prob	Type
0.111	0.049	Initial Uptake slope(in) *high* & uptake skewness *low* & irregularity *medium to high*	C	0.614	T1w dynamic & T1w morphology
0.031	0.06	dispersion *not low* & 3rd post-SE *high* & predicted LMSIR *very low*	C	0.548	dispersion & single-time-Enh & T2w

Freq = rule frequency, Err = rule error rate, Pred = rule prediction, Prob = rule probability, Type refers to the feature group of the rule.

**Fig 5:**
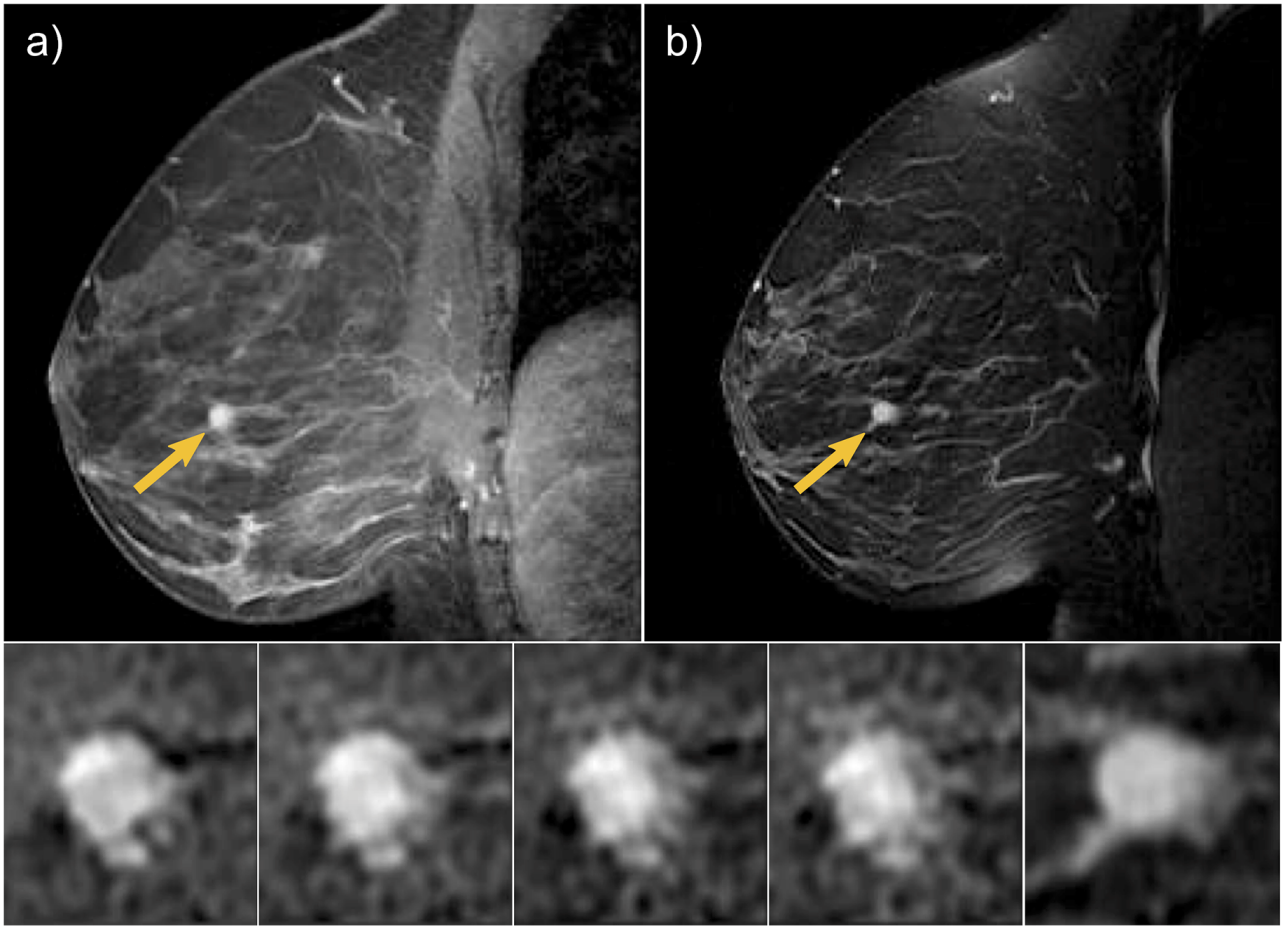
A benign fibroepithelial lesion with fibrocystic changes and hyperplasia in a 35-year old high risk woman. The lesion appeared as a circumscribed round mass, with smooth margins, rapid early enhancement and mild washout in delayed enhancement (BI-RADS score 4). a) T1w 4th post-contrast DCE-MRI. b) T2w MIR. c) zoomed progressive enhancement at each of the four post-contrast scans. d) zoomed lesion T2w appearance.

Fig 5 shows a benign fibroepithelial lesion with fibrocystic changes and hyperplasia in a 35-year old high-risk woman. The lesion appeared as a circumscribed round mass, with smooth margins, rapid early enhancement and mild washout in delayed enhancement (BI-RADS score 4). The appearance in T2w imaging was described as hyperintense. Table 5 reports the top two scoring rules that explained CAD classification as a benign lesion.

**Table 5:** Explanation of CAD classification output for lesion in Fig 5.

freq	err	condition	Pred	Prob	Type
0.057	0.032	Max radial gradient variance *very low* & sum average *medium to high* & 1st post-SE *not low* & T2w inverse difference moment *not low*	NC	0.505	T1w morphology & T1w texture & single-time-Enh & T2w
0.127	0.043	uptake skewness *low* & irregularity *very low* & dispersion *not low*	NC	0.424	T1w dynamic & T1w morphology & dispersion

Freq = rule frequency, Err = rule error rate, Pred = rule prediction, Prob = rule probability, Type refers to the feature group of the rule.

## Discussion and conclusion

This study demonstrated that relative T2w lesion intensity can be evaluated quantitatively from routine breast MRI imaging using a regression model and can be used to improve the performance of CAD for differential diagnosis of breast MRI lesions. AUC increased from 0.80 to 0.83 and this difference was statistically significant (adjusted p-value = 0.020). The inclusion of T2w texture, margin, and the predicted LMSIR resulted in an increase in specificity (0.75 to 0.80 or 5%) compared to a classifier that used only DCE-T1w features. At the optimal sensitivity of 0.70 with only DCE-T1w features (middle point of the solid-orange cross in Fig 3), the plots demonstrate higher specificity with the use of T2w features (dotted-dark blue curve). However, since these are points on the ROC curve they suffer from the trade-off between sensitivity (that rises as we move up the curve) and specificity (that drops as we move right). On the other hand, the AUC allows to quantitatively compare the performance of the different classifiers and to perform statistical hypothesis testing to determine if they are statistically different, independently of a chosen operating threshold.

Automatically generating a feature of relative T2w lesion intensity is potentially important in the application of CAD algorithms to multicenter studies, in which differences in scanner hardware and software can cause CAD algorithms based on T2w absolute MRI intensities to underperform. The proposed predictive-based relative T2w lesion intensity feature attempts to overcome this challenge and to extend the applicability of CAD systems. Although the predicted LMSIR feature used in group-4 model is derived from the same T2w features such as texture and margin as are used in group-2 model, our results suggest that incorporating additional information about the lesion intensity relative to the muscle signal during training of the highly non-linear LMSIR regression model results in a marginal improvement in AUC. In addition, our proposed method has the advantage of not requiring the measurement of muscle intensity for any future case; assessing relative T2w lesion intensity only requires the proposed regression model, without the need to measure an additional reference region in the muscle.

In clinical practice, BI-RADS T2w categories are selectively reported by the radiologist, suggesting that the evaluation of T2w imaging is relevant in the differential diagnosis of certain lesions but not others. This observation has been previously documented in the literature, but this expert knowledge has not been incorporated in CAD. Our results found that a CAD system also finds the additional information from T2w imaging selectively relevant, as illustrated by the presence of T2w features among the top scoring rules explaining classification results in 25 out of 70 fibrocystic lesions but only in 9 out of 136 invasive ductal carcinomas. Our results also indicate that the inclusion of relative T2w lesion intensity offers improved discrimination and generalization, as opposed to the absolute T2w intensity. Other CAD systems previously reported use classifications based on the BI-RADS classification lexicon. In [26], computer-extracted features are linked to dynamic and morphologic descriptors defined in the BI-RADS MRI atlas, using a separate classifier that estimates a morpho-dynamic index (MDI) of the probability of malignancy. Similarly, linking the automated feature of relative T2w lesion intensity proposed in this study to the corresponding BI-RADS T2w lexicon category could be addressed with an additional linking classifier.

T2w morphology and topology could offer additional discrimination but were not evaluated which is a limitation of this study. Although it has been suggested that analysis of T2w morphology and topology can aid in the recognition of intraductal fluid or cysts [27], the delineation of lesion contours in T2w scans might not be possible in the absence of lesion intensity. Our approach has the advantage of requiring the lesion contour from DCE-MRI only, and T2w texture and margin analysis to derive a model of relative T2w lesion intensity and produce diagnostically useful T2w CAD features.

One limitation of this study is that both mass and nonmass-like lesions were included, but the analysis was not done separately according to enhancement type. It is known that T1w kinetic analysis is not equally effective for both mass and nonmass-like enhancements [13, 28], and therefore is also possible that the same T2w features maybe not be equally discriminative for both enhancement types. Future work could carry out CAD lesion characterization according to enhancement type both on T1w and T2w imaging on a larger group of lesions.

Since high predictive performance is one of the pre-requisites for the applicability of a CAD system to human reading studies, results of this study are encouraging. The assistance of CAD in screening breast MRI is still a matter of further research, but we can argue in favor of the implications of a significant increase in CAD predictive performance. Increasing sensitivity while maintaining high specificity will result in an increase of breast cancers detected by screening breast MRI, and increasing specificity while maintaining high sensitivity will result in a reduction of false-positive results with screening breast MRI.

CAD outcomes can be positive (classifying the lesion as malignant) or negative (classifying the lesion as benign). Our CAD sensitivity is estimated as the percentage of diagnosed breast cancer lesions that had a positive CAD diagnosis and CAD specificity as the percentage of diagnosed benign lesions that had a negative CAD diagnosis. If we use the OBSP sensitivity of 86.1% [2] as the minimum operating sensitivity for CAD, the resulting CAD specificity is 53.1% for CAD lesion characterization based on T1w DCE-MRI only, and specificity increases to 61.3% with the inclusion of T2w MRI in CAD lesion characterization. This increase in specificity is equivalent to a reduction of 8 false-positive results per 100 women screened with breast MRI who are found to not have cancer.

In conclusion, our results showed that CAD predictive performance improved significantly with the introduction of T2w MRI lesion characterization. This improvement is important because CAD can potentially increase both sensitivity and specificity of screening breast MRI by aiding medical professionals.

## Supporting information

S1 Table List of computer-extracted lesion features from T1w DCE-MRI and T2w MRI. (DOCX)
