# Supplementary material for: Using quantitative features extracted from T2-weighted MRI to improve breast MRI computer-aided diagnosis (CAD)
Source: PLoS One. 2017 Nov 7;12(11):e0187501. doi: 10.1371/journal.pone.0187501 (PMC5675400; doi:10.1371/journal.pone.0187501)
Supplement: S1 Table — (DOCX) [file pone.0187501.s001.docx]

**Category: T1w DCE Kinetic features**

| # | Feature name | | Feature description | |  |
| --- | --- | --- | --- | --- | --- |
| The following model can be used to fit the relative signal changes after contrast injection:  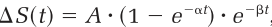  Where A is the upper limit of the signal intensity, alpha(min^-1^) is the rate of signal increase, beta(min^-1^) is the rate of the signal decrease during washout. The goodness of fit parameter R^2^ is calculated for each lesion to guarantee goodness of fit. Other details of the empirical model can be found in [1]. | | | | | |
| K1 | **Relative signal change amplitude** | the upper limit of the signal intensity change in the eq. above | | | |
| K2 | **alpha(min^-1^)** | the rate of signal increase | | | |
| K3 | **beta(min^-1^)** | rate of the signal decrease during washout | | | |
| K4 | **Initial area under curve (AUC)** | **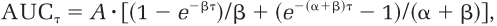**  where tao=time at first-post contrast acquisition, is the time over which AUC is taken. | | | |
| K5 | **Initial slope of enhancement (Slope_ini)** | 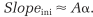  Initial slope of the kinetic curve can be calculated by taking the derivative of **AUC** | | | |
| K6 | **Time to peak of enhancement (Tpeak)** | **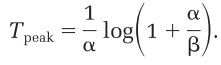**  The time at which the kinetic curve reached peak  Taken from setting the derivative of AUC equal to zero. | | | |
| K7 | **Enhancement curvature at peak (Kpeak)** | **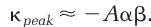**  The curvature at the peak of enhancement was calculated from the definition of curvature formula at time of Tpeak | | | |
| K8 | **Signal enhancement ratio (SER)** | 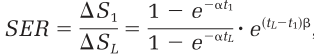  The signal intensity change at first-post contrast time (t1) relative to the last-post contrast time (tL) | | | |
| K9 | **maximum uptake of lesion average enhancement (FII,1)** | Based on 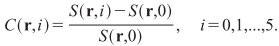  Where S(r,i) is the voxel intensity at **ith**-post contrast time, S(r,0) at pre- contrast time, and **r** ranges over voxels inside the lesion volume. As proposed in [2].  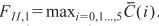 | | | |
| K10 | **peak location (FII,2)** | ith-post contrast time (i) at which the maximum enhancement occurs | | | |
| K11 | **uptake rate (FII,3)** | Ratio of maximum uptake to peak time: 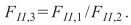 | | | |
| K12 | **washout rate (FII,4)** | Ratio of the difference between maximum uptake and last-post contrast uptake:  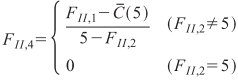 | | | |
| Variance of uptake: Variance V(i) the computation of the variance of the voxel intensities at the ith-post contrast time (i) over r, where r ranges over voxels inside the lesion volume. As proposed in [3].  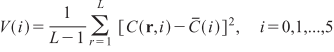 | | | | | |
| K13 | **maximum variation of enhancement (FIII,1)** | | | Maximum spatial variance of enhancement  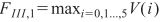 | |
| K14 | **peak of variance of enhancement (FIII,2)** | | | **ith**-post contrast time **(i)** at which the maximum variation of enhancement occurs | |
| K15 | **enhancement-variance increasing rate (FIII,3)** | | | Ratio of maximum variation of enhancement to peak time: 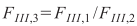 | |
| K16 | **enhancement-variance decreasing rate (FIII,4)** | | | Ratio of the difference between maximum variation of enhancement and last-post contrast variation of enhancement:  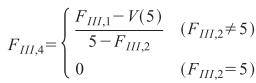 | |
| K17 | **Enhancement-variance at the first-post contrast frame (FIII,5)** | | | V(1), reveals the uptake inhomogeneity at the early phase of uptake. | |

**Category 2: Dispersion of T1w DCE-MRI**

| # | Feature name | | Feature description |  |
| --- | --- | --- | --- | --- |
| Based on calculating *k-means* clusters (n=20) of the lesion voxels, and grouping voxels in the resulting clusters | | | | |
| PC1-80 | | **Lesion cluster relative signal enhancement** | 20 measurements of relative Signal enhancement per **ith**-post contrast time, $i=1,..,4$ | |
| D1-D20 | | **spatial dispersion of signal enhancement** | Calculate the Euclidean distance between each of the cluster centroids and the lesion center to obtain measures of lesion dispersion. | |

**Category 3A: T1w DCE Morphology**

| # | Feature name | | Feature description |  |
| --- | --- | --- | --- | --- |
| Based on calculating a shell (Fm)—three voxels thick—centered on the surface of the lesion.  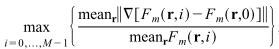, where 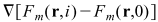 is the set of voxel-value gradients at the margin of the lesion at each ith-post contrast time. Computation of the spatial gradient is accomplished in 3D by convolution with the components of a 3x3x3 Sobel filter in three orthogonal directions. | | | | |
| Mt1w1 | | **Max Margin Gradient** | Maximum margin gradient (above equation) | |
| Mt1w2 | | **Time to Max Margin Gradient** | **ith-**post contrast time point to Maximum margin gradient  (ith to max of Mt1w1) | |
| Mt1w3 | | **Max Variance of Margin Gradient** | Maximum variance of margin gradient =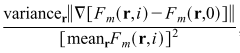 | |
| Mt1w4 | | **Time to Max Variance of Margin Gradient** | **ith-**post contrast time point to Maximum Variance of Margin Gradient (ith to max of Mt1w3) | |
| Mt1w  5-11 | | **Moments of the Fm shell** | Minimum, maximum, mean, median, variance, skewness, and kurtosis of the three voxels thick shell (**Fm**) at the surface of the lesion | |
| Mt1w12 | | **Circularity** | 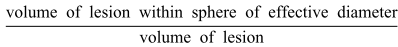 | |
| Mt1w13 | | **Irregularity** | **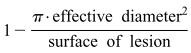**, where effective diameter =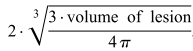 | |
| Radial gradient histogram analysis (RGH): Histogram of the dot product between voxel-value gradients and lines intersecting a single point at the center of the lesion (i.e lines in a radial direction rc)  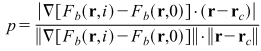 Fb is set of voxel-value gradients, and r ranges over voxels at the margin of the lesion volume. As proposed in [2]. | | | | |
| Mt1w14 | | **Maximum Average of RGH** | Max average of Radial gradient histogram across **ith-**post contrast time points. | |
| Mt1w15 | | **Time to Max Average of RGH** | **ith-**post contrast time point to Max average of Radial gradient histogram | |
| Mt1w16 | | **Maximum Variance of RGH** | Max Variance of Radial gradient histogram across **ith-**post contrast time points. | |
| Mt1w17 | | **Time to Max Variance of RGH** | **ith-**post contrast time point to Max Variance of Radial gradient histogram | |
| Lesion Edge Sharpness: As proposed in [4].  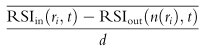a function of position (ri) and time (t); ri is a voxel position: RSI*in* in inner ring of the margin, RSI*out* in outer ring of the margin.  n(ri) is the six-connected three-dimensional neighborhood of ri.  d is a normalization term | | | | |
| Mt1w18 | | **Mean of Lesion Edge**  **Sharpness** | Average of lesion edge sharpness across post-contrast time points | |
| Mt1w19 | | **Variance of Lesion Edge**  **Sharpness** | Variance of lesion edge sharpness across post-contrast time points | |

**Category 3B: T2w Morphology**

| # | Feature name | | Feature description |  |
| --- | --- | --- | --- | --- |
| Similarly as for T1w, T2w margin gradients are calculated based on Fm, a shell—three voxels thick—centered on the surface of the lesion.  $\boldsymbol{mean}_{\boldsymbol{r}}\left\Vert{\boldsymbol{\nabla}\boldsymbol{F}}_{\boldsymbol{m}}\boldsymbol{(r)} \right\Vert$, where ${\boldsymbol{\nabla}\boldsymbol{F}}_{\boldsymbol{m}}\boldsymbol{(r)}$ is the set of voxel-value gradients at the margin of the lesion. Computation of the spatial gradient is accomplished in 3D by convolution with the components of a 3x3x3 Sobel filter in three orthogonal directions. Note that on T2w imaging there’s no multiple time points i, so only one value is computed from the T2w lesion VOI. | | | | |
| Mt2w1-7 | | **Moments of the Fm shell** | Minimum, maximum, mean, median, variance, skewness, and kurtosis of the three voxels thick shell (**Fm**) at the surface of the lesion | |
| Radial gradient histogram analysis (RGH): Histogram of the dot product between voxel-value gradients and lines intersecting a single point at the center of the lesion (i.e lines in a radial direction)  Fb is set of voxel-value gradients, and r ranges over voxels at the margin of the lesion volume. | | | | |
| Mt2w8 | | **average of RGH** | average of Radial gradient histogram | |
| Mt2w9 | | **variance of RGH** | variance of Radial gradient histogram | |

**Category 4: T1w DCE and T2w Texture**

| # | Feature name | | Feature description |  |
| --- | --- | --- | --- | --- |
| 3D texture features based on the non-directional grey-level co-occurrence matrix (GLCM) of voxel-pair statistics. We extracted a non-directional GLCM by summing 13 directional GLCMs. As proposed in [5]. For T1w texture a GLCM is computed at each of the post contrast volumes. From T2w texture features only one GLCM is computed from the T2w volume. Then, from the resulting non-directional GLCM matrix, 11 voxel-pair statistics measures are derived: | | | | |
| T1 | | **Energy or Angular second moment** | | |
| T2 | | **Contrast** | | |
| T3 | | **Correlation** | | |
| T4 | | **Variance** | | |
| T5 | | **Inverse difference moment** | | |
| T6 | | **Sum average** | | |
| T7 | | **Sum variance** | | |
| T8 | | **Sum entropy** | | |
| T9 | | **Entropy** | | |
| T10 | | **Difference variance** | | |
| T11 | | **Difference entropy** | | |

**References:**

[1] S. A. Jansen, X. Fan, G. S. Karczmar, H. Abe, R. A. Schmidt, M. Giger, and G. M. Newstead, “DCEMRI of breast lesions: is kinetic analysis equally effective for both mass and nonmass-like enhancement?,” *Med. Phys.*, vol. 35, no. 7, pp. 3102–3109, 2008.

[2] W. Chen, M. L. Giger, L. Lan, and U. Bick, “Computerized interpretation of breast MRI: Investigation of enhancement-variance dynamics,” *Med. Phys.*, vol. 31, no. 5, pp. 1076–1082, Apr. 2004.

[3] K. G. A. Gilhuijs and M. L. Giger, “Computerized analysis of breast lesions in three dimensions using dynamic magnetic-resonance imaging,” *Med. Phys.*, vol. 25, no. 9, pp. 1647–1654, 1998.

[4] J. E. D. Levman and A. L. Martel, “A Margin Sharpness Measurement for the Diagnosis of Breast Cancer from Magnetic Resonance Imaging Examinations,” *Acad. Radiol.*, vol. 18, no. 12, pp. 1577–1581, Dec. 2011.

[5] W. Chen, M. L. Giger, H. Li, U. Bick, and G. M. Newstead, “Volumetric texture analysis of breast lesions on contrast-enhanced magnetic resonance images,” *Magn. Reson. Med.*, vol. 58, no. 3, pp. 562–571, Sep. 2007.
